# Reframing neuroergonomics in an evolutionary and active inference context

**DOI:** 10.3389/fnrgo.2026.1796721

**Published:** 2026-05-07

**Authors:** Farah I. Corona-Strauss, Jonas Vibell, Alexander L. Francis, Martina Lehser, Sebastian M. Markert, Daniel J. Strauss

**Affiliations:** 1 Systems Neuroscience & Neurotechnology Unit, Faculty of Medicine, Saarland University & School of Engineering, htw saar, Homburg/Saar, Germany; 2 Center for Digital Neurotechnologies Saar, Saarland University and htw saar, Saarbrücken and Homburg/Saar, Germany; 3 Brain and Behavior Laboratory, Department of Psychology, University of Hawai'i at Manoa, Honolulu, HI, United States; 4 Speech Perception and Cognitive Effort Lab, Department of Speech, Language and Hearing Sciences, Purdue University, West Lafayette, IN, United States

**Keywords:** active inference, evolution, multisensory processing, neuroergonomics, sensory conflict, sensory strain

## Abstract

Everyday situations, such as feeling nauseous in virtual-reality environments or getting dizzy when reading as a car passenger, reveal how easily our senses can become confused when modern technology disrupts the innate relationship between the physical environment and human sensory systems. Such disruptions expose the vulnerability of the human senses to conflicting input arising in technologically altered environments. Even in the absence of direct sensory conflict, in complex technological settings such as digital factories and modern operating rooms, the convergence of multiple competing stimuli within and across sensory modalities further amplifies sensory load and cognitive strain. The common denominator of all such problems is that our ancient sensory processing and perceptual systems do not fit well with the technological world we have created. This evolutionary mismatch is already significant, but it will become even more critical as mixed reality concepts and advanced digital technologies integrate more deeply into our daily lives. Focusing on sensory mismatch and sensory strain as two significant ramifications of the Anthropocene, we reframe neuroergonomics in an evolutionary and active inference context. Our reframing argues that neuroergonomics must prioritize technology design that respects evolutionarily tuned priors, and should additionally deploy measured epigenetic, gene–culture, and learning-driven interventions as complementary levers to support adaptive change. Thus, we highlight the importance of aligning and grounding neuroergonomic design with the human sensory system according to constraints and affordances defined by human evolutionary history.

## 1 Introduction

The Anthropocene, a term referring to our current era within the geological epoch of the Holocene, has reconfigured landscapes and their multisensory signatures: changes in land-use, infrastructure and material culture alter auditory, visual, somatosensory and chemosensory properties of environments across nested spatial and temporal scales (Pijanowski et al., 2011; Schafer, 1977). Contemporary landscapes carry layered acoustic emissions from people, vehicles and industry; transformed visual texture from built structures and lighting; altered somatosensory affordances via engineered surfaces and vibration transmission; and reworked olfactory baselines from novel emissions and disrupted biogenic sources. These parallel changes increase multisensory background “noise”, elevate cross-modal masking and source ambiguity, and shift the ecological statistics to which human perceptual systems are adapted (Pijanowski et al., 2011; Stein, 2012).

Each modality supplies complementary information for ecological active inference: audition for distant temporal events, vision for spatial layout and object identity, touch for substrate vibration and contact confirmation, and olfaction for slow, affect-rich chemical context (Ghazanfar and Schroeder, 2006; Herz, 2004). Landscape change is heterogeneous in scale and time: broad land-cover conversion alters ambient spectral and olfactory baselines over seasons to decades, while localized infrastructure (roads, industrial sites, ventilation, signage) creates point sources with steep spatial gradients and rapid temporal fluctuations that dominate local perception (Pijanowski et al., 2011; Kang and Schulte-Fortkamp, 2016).

Multisensory interactions are asymmetric and context dependent: congruent cues reduce uncertainty and improve detection and localization, whereas asynchronous or competing cues induce false binding, attentional capture and degraded inference (Calvert et al., 2004; Stein, 2012). Common landscape mismatches such as visually natural scenes paired with persistent industrial acoustic or vibratory signatures, or engineered lighting regimes that conflict with olfactory and acoustic baselines, violate ecological priors and can alter navigation, habitat choice, stress responses and place attachment (Pijanowski et al., 2011; Herz, 2004).

**Figure 1:**
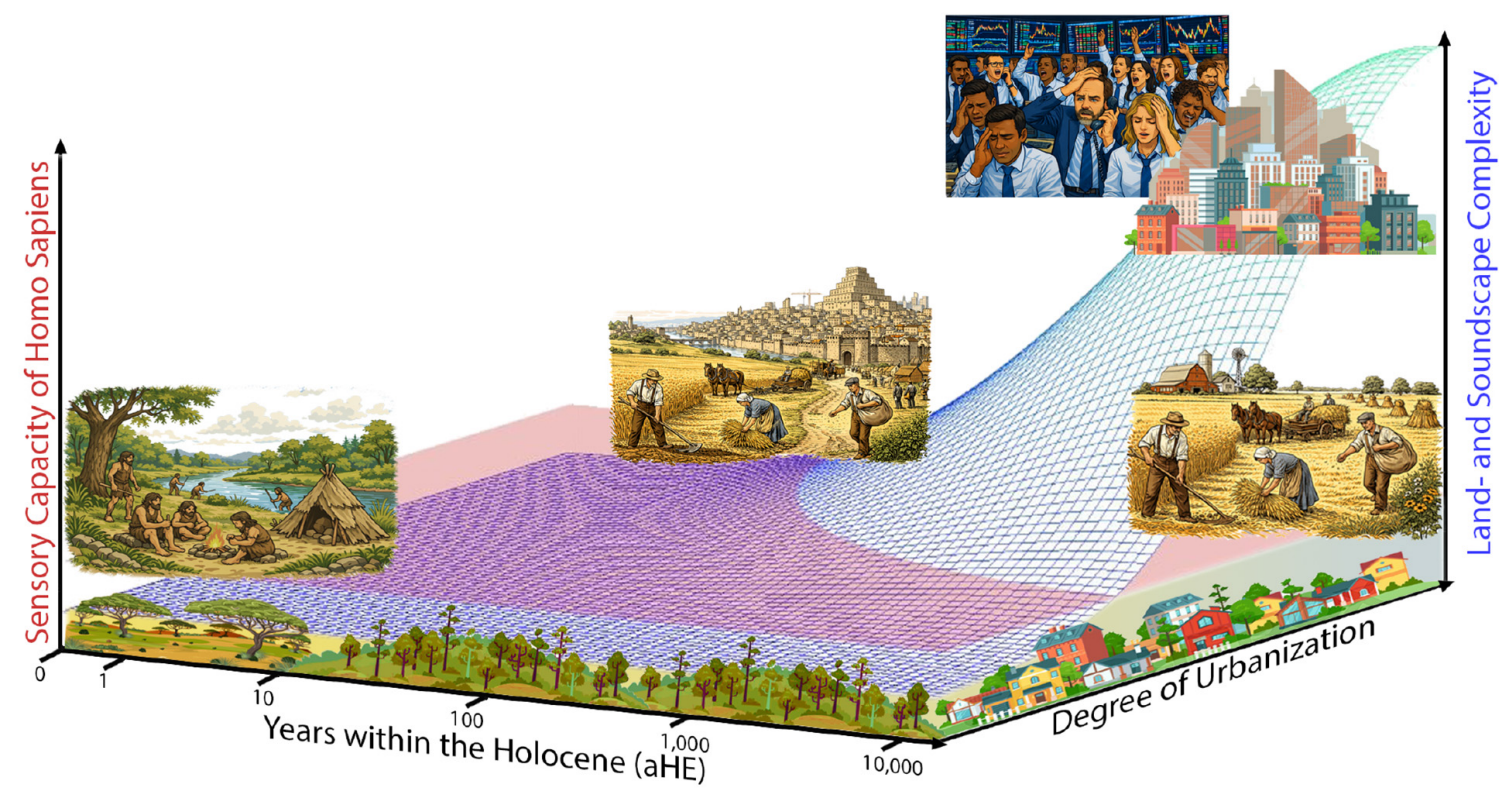
Conceptual sketch contrasting the changing complexity of land- and soundscapes with human sensory capacity throughout the Holocene (x-axis; logarithmic scaling in years, aHE). Sensory capacities that are likely to be more genetically entrenched are assumed to remain essentially constant over the Holocene (left y-axis; the peach-colored plane illustrates this assumed constancy). In contrast, the complexity of land- and soundscapes (right y-axis; blue-to-green gradients) increases markedly with rising degrees of urbanization in recent years (z-axis), especially since industrialization.

Crucially, cultural and technological change has accelerated the transformation of multisensory environments far faster than biological evolution can accommodate. In Pleistocene environments, when many human perceptual priors were shaped, attending to non-human signals (predator calls, prey movement, wind, substrate vibration) was central to survival and coordinated action (Tooby et al., 1992). Rapid cultural niche construction has since altered multisensory statistics and affordances within mere generations, producing an evolutionary mismatch: integrative mechanisms tuned to ancestral statistics now operate in worlds with novel spectral content, temporal dynamics and engineered affordances (Boyd and Richerson, 1985; Pijanowski et al., 2011). As a result, modern multisensory urban landscapes may be both quantitatively richer and qualitatively different from those that shaped our perception. Cultural evolution has outpaced biological evolution by far, creating mismatches between evolved multisensory priors and present-day informational demands, see Figure 1.

By foregrounding sensory conflict and sensory strain in this perspective, we place neuroergonomics in an evolutionary perspective that recognizes how the multisensory landscapes of the Pleistocene differ from those of the Anthropocene and how this divergence affects perception, behavior and wellbeing.

## 2 Spatiotemporal symphony of the senses

**Figure 2:**
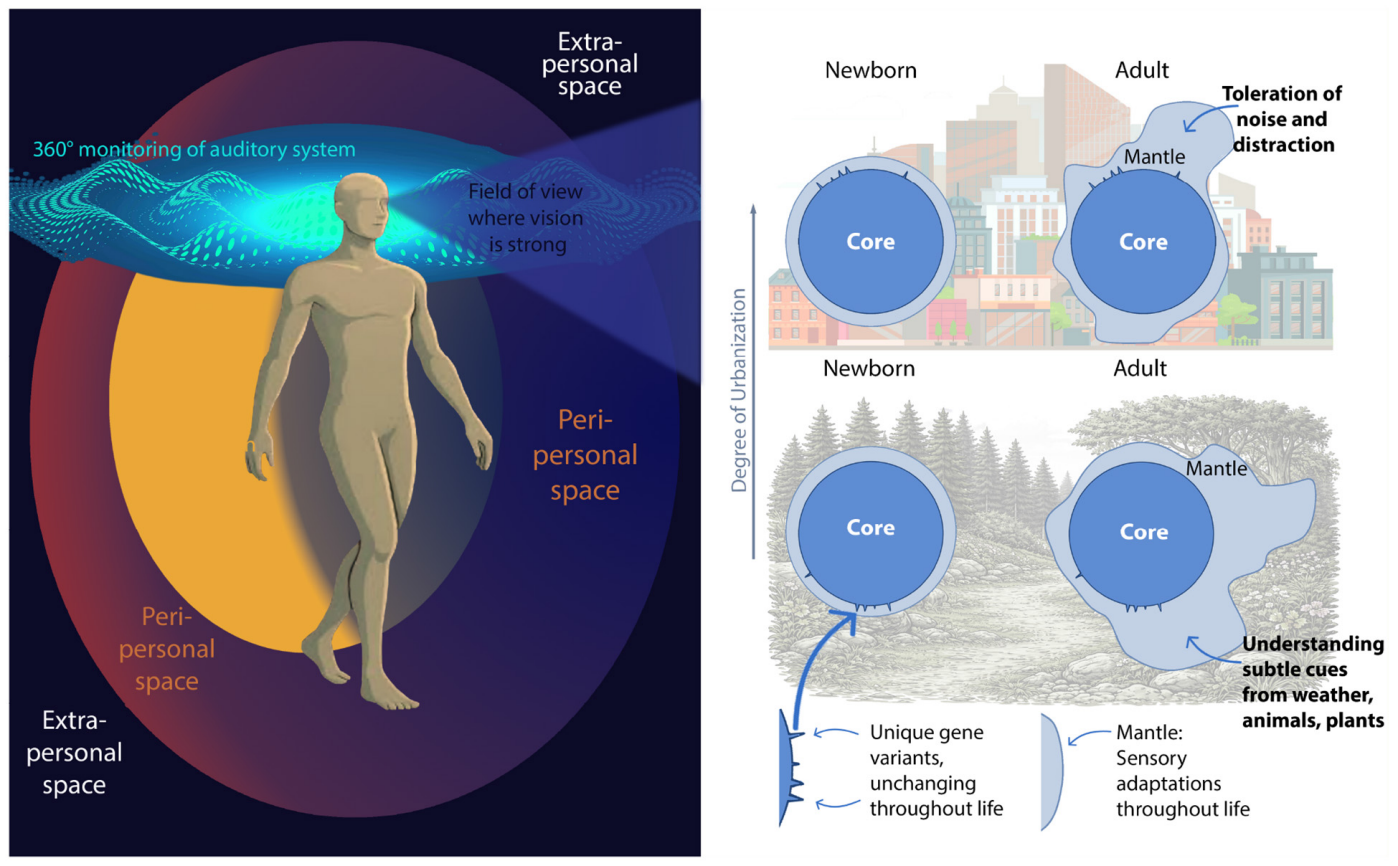
**(Left)** The spatial dynamics of genetically entrenched auditory, visual, and somatosensory systems: the 360° acoustic monitoring of the auditory system, sharp visual resolution within the field of view, and the peripersonal space with its strong visuo-tactile integration (see Rabellino et al., 2020) of humans. **(Right)** A speculative and heuristic illustration of the distinction between two conceptual layers that may affect human perception and cognition: The ”core” shared by the whole species that features only small individual differences and that changes over evolutionary time scales, and the ”mantle” of individual differences acquired over a life-time. Two humans in very different environments will begin life very similar and then their mantles diverge through modulatory influences.

We present a heuristic framing in which human perception is described as arising from the interaction between relatively stable, evolutionarily biased mechanisms (the “core”) and a set of modulatory, context-sensitive processes (the “mantle”). We adopt the “core” vs. “mantle” terminology as a pragmatic heuristic to capture differing degrees of stability and plasticity; these labels are intended to guide empirical inquiry rather than to assert a strict, immutable partition (see Figure 2, right), and they highlight different degrees of stability and plasticity as well as different functional emphases (species-typical biases vs. context-dependent modulation), rather than implying a strict anatomical or ontological partition. The “core” denotes *species-typical* biases that shape baseline priors for multisensory inference; the “mantle” denotes plastic, experience-dependent processes of *individual humans* (including learning, neuromodulatory gating, myelin and synaptic plasticity, and environmentally mediated epigenetic regulation) that tune those priors across development and context. Contemporary evidence emphasizes continuous, bidirectional interactions between predisposition and experience (e.g., see Holtmaat and Svoboda, 2009; Ribic, 2020); accordingly, the boundary between “core” and “mantle” is porous and dynamic, and the terms should be read as pragmatic distinctions to aid exposition rather than as rigid scientific demarcations.

Many traits that we describe as part of the “core” reflect adaptations shaped in ancestral environments and continue to bias perception today. Given that evolution is extremely slow compared to the development of human culture and technology, it is reasonable to assume that a prehistoric *Homo sapiens* during the Upper Paleolithic (about 50,000 BCE) had a genetic makeup broadly similar to that of modern humans. From hunter-gatherers to ancient civilizations to contemporary societies, many species-typical tendencies persist. For example, humans are highly sensitive to unexpected sounds outside the visual field (Mobbs et al., 2015; Strauss et al., 2020; Olszanowski et al., 2023; Schroeer et al., 2023, 2024), to peripheral motion (Yantis and Jonides, 1984), and to social-affective cues in faces (Taubert et al., 2017); tendencies that plausibly reflect priors shaped by past ecological demands (Tooby et al., 1992; Dosen and Ostwald, 2016; Corbetta and Shulman, 2002). These predispositions are not immutable: they interact continuously with learning and other modulatory mechanisms described below, so that evolutionary heritage and lifetime experience jointly shape perceptual inference and behavior.

### 2.1 The core: genetically entrenched concepts

Human multisensory architecture is anchored by a set of evolutionarily conserved, genetically biased mechanisms that provide the primary scaffold for perception and attention. These mechanisms represent the “species-level defaults,” the “core” of sensory integration in humans, shaping how information is weighted and combined across modalities. Several key features illustrate this foundational tier. First, 360° auditory monitoring provides continuous, omnidirectional surveillance of the environment, enabling detection of salient events outside the visual field and supporting rapid orienting toward potential threats or opportunities (Ignatiadis et al., 2025; Strauss et al., 2020; Olszanowski et al., 2023; Strauss et al., 2025). Second, early-developing vestibular–proprioceptive coupling stabilizes posture and establishes spatial reference frames, ensuring that movement and orientation are grounded in reliable bodily signals (Merfeld et al., 1999). Third, privileged foveal visual processing supplies high-resolution spatial detail in human vision, allowing fine-grained discrimination of objects and events within the central field (Spencer and Polich, 1999; Findlay and Gilchrist, 2003). Fourth, body-centered peri-personal multisensory mapping links touch, vision, and action to create an integrated representation of the space immediately surrounding the body (Noel et al., 2018). See Figure 2 (left) for a spatial mapping of these senses, which is pertinent to neuroergonomic design. Finally, the spatiotemporal symphony of the rapid cross-modal orienting responses in humans, involving these senses, is tuned to salient, behaviorally relevant events, enabling swift shifts of attention when survival or task demands require it (Calvert et al., 2000; Spence et al., 1998; Calvert et al., 2004; Strauss et al., 2024; Thinnes et al., 2025).

Of note, many features we describe as part of the “core” are plausibly shaped by evolutionary pressures and therefore may bias perception across contexts, but these tendencies should be treated as probabilistic predispositions that interact continuously with cultural and developmental influences. This means that there is a relationship between perception and cognition and our genes. Such bridges between psychology and genetics are notoriously difficult to reveal, but some clues can be found by harnessing neurodiversity. Outliers in cognitive tasks could also be outliers with respect to their gene variants, thus revealing the links (Parasuraman and Rizzo, 2007; Parasuraman, 2013). For example, people who show exceptional performance in reaction time, working memory, or visual attention were found to exhibit special genetic variants for certain proteins involved in brain activity (Greenwood et al., 2012, 2009; Parasuraman et al., 2012; Lancaster et al., 2017). These findings offer first glimpses into how our perception and thinking are influenced by our genes. But, of course, there is not only nature, but also nurture. The next section is concerned with influences other than genes, that shape us individually during our lifetimes.

### 2.2 The mantle: genetic, epigenetic, and experience-driven modulations

A distinct, superimposed set of influences, here grouped as modulatory processes, continuously tune, amplify, or attenuate the genetically entrenched scaffold across development, populations, and contexts, see Figure 2 (right). This category subsumes (1) epigenetic regulation, which gates gene expression and sensitive-period timing in response to early environment and activity; (2) gene–culture co-evolution, where persistent cultural practices and sensory ecologies create selection pressures that shift population-level priors over generations; and (3) individual learning and adaptation, spanning rapid synaptic reweighting, intermediate consolidation, and long-term cortical remapping (Ashe and Colot, 2021; Hawks et al., 2007; Gintis, 2011; Boduroglu and Shah, 2017; Jurkat et al., 2020; Laland et al., 2010). Epigenetic mechanisms (DNA methylation, histone modification, non-coding RNAs) mediate how environmental inputs and activity patterns alter the expression of genes relevant for synaptic plasticity and sensory system development, thereby modulating sensitive periods and lifelong responsiveness (Ashe and Colot, 2021; Kleim and Jones, 2008). This is why although identical twins begin life with the same genetic sequence, they diverge over time due to epigenetic modifications. Factors like diet, stress and lifestyle will influence how strongly genes are expressed, resulting in different health and disease susceptibility outcomes such as skin cancer or type 2 diabetes. Gene–culture feedback loops operate over generations by changing the sensory contexts in which selection acts (e.g., acoustic, visual, dietary, and tool-use environments), producing slow shifts in population priors that coexist with rapid, individual-level plasticity (Hawks et al., 2007; Gintis, 2011; Laland et al., 2010; Boyd et al., 2013). A classic example of gene culture co-evolution is (loss of) lactose intolerance: While most mammals lose the ability to digest milk after weaning, human populations that adopted dairy farming favored mutations allowing lifelong lactase production making milk consumption a genetic and cultural adaptation.

Learning and adaptation operate on multiple time scales—fast trial-by-trial reweighting, medium-term consolidation of perceptual skills, and slow representational restructuring—and should be parameterized explicitly as context- and history-dependent adjustments to the baseline priors, with quantifiable uncertainty (Kleim and Jones, 2008; Boduroglu and Shah, 2017). Learning to play a musical instrument encompasses all the stages. In the fast learning phase, the player corrects trial by trial mistakes by adjusting finger movements. With repeated practice, skills consolidate and playing becomes smoother. Finally, slow learning appears over years as musicians restructure mental representations, moving from memorizing notes to understanding harmony and improvising.

## 3 Sensory conflict and strain in our modern life

This sections examines how the demands of contemporary environments challenge the coherence of our multisensory systems in neuroergonomics. Everyday technologies and immersive media frequently generate sensory conflict, where incongruent inputs across modalities disrupt perception and induce discomfort. Beyond conflict, modern contexts also impose sensory strain, the cumulative burden of sustaining attention and recalibrating precision weights under noisy or demanding conditions, see also (Karwowski 2006). To move beyond isolated accounts, this section culminates in Unifying Sensory Conflict and Strain Using Active Inference, offering a framework that integrates these phenomena into a single explanatory model of how the brain negotiates stability in complex, multisensory worlds.

### 3.1 Sensory conflict

Sensory conflict or sensory mismatch, defined as a violation of expected regularities in sensory input (Treisman, 1977; Bertolini and Straumann, 2016), has been a recurrent phenomenon since *Homo sapiens* began developing technologies to facilitate daily life, particularly in the context of mobility—from ships (seasickness) to automobiles (motion sickness). A common example is the experience of nausea when reading as a passenger in a moving car: the visual system conveys a state of rest, while the vestibular system detects motion, resulting in a conflict that induces discomfort. Converging evidence suggests that the only ”explanation” available to our ancient brain structures for such sensory mismatch is intoxication (Nalivaiko et al., 2014; Bertolini and Straumann, 2016; Schneider et al., 2022). Simulated interactive environments such as vehicle simulators and mixed reality worlds can push cybersickness to an extreme, either intentionally or unintentionally. Thus, they also provide excellent, highly controlled, multisensory contexts with which to study the origins, consequences, and individual differences in response to sensory conflict, allowing us to examine, for example, both general susceptibility and individual differences in habituation capacity to nausea (Laessoe et al., 2023; Buchheit et al., 2021; Schneider et al., 2022; Lehser et al., 2024). These phenomena illustrate that sensory conflict is not only a perceptual anomaly but also a deeply embodied experience (see Section 2): cognition is inseparable from the body's sensory systems and their interaction with the environment, e.g., see (Shapiro 2011) and (Wöstmann and Obleser 2026). Proprioception, the continuous sensing of body position and movement, is central here, as its alignment or misalignment with visual and vestibular cues can determine whether a situation feels stable or disorienting, see also (Limanowski and Friston 2020). In this sense, embodied cognition offers a powerful lens for understanding why conflicts across modalities can so profoundly shape perception, action, and affective state. Although such mismatches are widespread, their generality across sensory domains and contexts, as well as the universal coping and compensation strategies humans employ, remain largely unexplored. This gap underscores the importance of neuroergonomic approaches that explicitly integrate embodied and proprioceptive dimensions into the study of human–technology interaction, which is particularly critical for virtual reality and emerging metaverse concepts (Valori et al., 2020; Bayramova et al., 2021).

### 3.2 Sensory strain

The novel environments afforded by the Anthropocene, increasing exponentially in number and variety, exert profound effects on human attention and the allocation of perceptual and cognitive resources within our digital, information-driven society (Stevens, 2013; Gazzaley and Rosen, 2016; Citton, 2017). Contemporary life is characterized by AI-driven systems, digital media platforms, and global networks of individuals competing for attentional engagement. When designing these attention-demanding technologies, the evolutionary foundations of the human attentional system, shaped under the ecological constraints of hunter-gatherer environments, are frequently overlooked. In environments where humans collaborate with machines such as Internet-of-Things (IoT) factory infrastructures or highly digitized operating rooms (Sendelbach, 2012), attention-grabbing stimuli often attempt to organize communication with the machines. However, even when optimized for a single device or interaction modality, the simultaneous presence of multiple machines and overlapping stimuli can generate a cacophony of alerts that induce strain. Moreover, the abundance of stimuli signaling opportunities (real or illusory) presented by our digital companions negatively impacts our mental health (e.g., see Gazzaley and Rosen, 2016; Citton, 2017). From an evolutionary perspective, attentional challenges remain insufficiently studied, and current approaches to neurergonomic “attention assistance” technologies within regulatory frameworks are still underexplored. Future research should investigate how design and regulation can integrate evolutionary insights into attentional support technologies, ensuring that digital systems align more closely with human cognitive capacities, whether genetically entrenched (Section 2.1) or modulatory (Section 2.2).

## 4 Unifying sensory conflict and strain using active inference

Within a multimodal framework, sensory conflict and strain can be fruitfully modeled as natural expressions of active inference under the Free Energy Principle (Friston, 2010). Sensory conflict or mismatch arises whenever different modalities deliver incongruent information, producing prediction errors that signal that the brain's generative model is insufficient to explain the current environment. For instance, when seeing but not feeling motion in virtual reality setups, the conflict with deeply entrenched physical regularities in the brain's predictive model evokes the “full body response” of cybersickness, see Section 3.1. While for sensory conflict the connection to active inference and its generative brain-modeling approach is direct (see also Limanowski and Friston, 2020), it is more nuanced for sensory strain. Sensory strain reflects the adaptive, resource-intensive regulation required to stabilize inference under uncertainty: reallocating precision and attentional gain across modalities and spatiotemporal scales to sustain goal-directed perception and action in noisy, dynamic environments. This has recently been analyzed for the auditory modality in (Strauss et al. 2025), using the unified exogenous- and endogenous-attention framework from (Parvizi-Wayne 2024). In particular, earlier models of attentional effort have been mapped to the free energy minimization framework that can be extended to multiple modalities (Strauss and Francis, 2017; Strauss et al., 2024). In a generative spatiotemporal scale space (Strauss et al., 2025), strain appears as coordinated control between the evolutionarily entrenched core (fast, local predictions and rapid exogenous adjustments) and higher, slower layers (abstract, forward-looking predictions and endogenous, reasoning-based loops). Precision-weight optimization determines which information streams are amplified or suppressed at each scale, depending on reliability, priors, and current goals (Feldman and Friston, 2010; Clark, 2013; Parvizi-Wayne, 2024; Strauss et al., 2025). Within the active-inference framework, minimizing uncertainty directly motivates the deployment of attentional effort (Sarter et al., 2006; Strauss and Francis, 2017) required to navigate sensory strain (see Feldman and Friston, 2010; Strauss et al., 2025). Operationally, this involves model updating, precision reweighting, and action/attention within an active inference framework, where attentional gain and noise suppression mechanisms enhance attended objects along the sensory pathways, and learned experiences modulate selection policies over time (Friston, 2010; Parr et al., 2022; Strauss et al., 2025). This perspective offers a concrete instantiation of this regulation: precision weights bias selection toward the target stream and away from distractors, integrating context and priors to maintain coherent tracking under load (Desimone and Duncan, 1995; Shinn-Cunningham, 2008; Trenado et al., 2009; Strauss et al., 2010). Strain, in this sense, is a graded expression of how much precision must be reallocated across the scale space to keep inference stable, increasing when distractor salience rises or when target predictability falls (Grossberg, 2005; Strauss and Francis, 2017; Parvizi-Wayne, 2024; Strauss et al., 2025). It also reflects the cost of maintaining long-horizon beliefs (e.g., conversational goals) against short-horizon exogenous overrides, with precision weights dynamically re-tuned as the (sensory) scene evolves (Clark, 2013; Parvizi-Wayne, 2024); see (Strauss et al. 2025) for a specific example listening scenario. Because precision is context- and also experience-dependent (see Section 2.2), identical scenes can induce different strain profiles across individuals, as priors and learned contingencies shape the weighting of candidate streams and the thresholds for reallocation (Trenado et al., 2009; Strauss et al., 2010). Ultimately, sensory strain marks the ongoing effort of a unified, active inference system to minimize immediate and future surprise by continuously tuning precision across spatiotemporal scales by balancing rapid exogenous responses with sustained endogenous control to preserve perceptual stability in complex multisensory settings.

## 5 Implications for neuroergonomics

From this perspective, we argue that neuroergonomic design (Karwowski et al., 2003; Parasuraman, 2003; Karwowski, 2006; Parasuraman and Wilson, 2008; Salvendy and Karwowski, 2021) should adopt a first-principles approach to sensory processing, asking what environments human perceptual systems are originally built for. Such an approach provides guidance on how best to support our senses in modern contexts and helps to clarify how all our senses can be used optimally to transfer information between humans, machines, and computers. Neuroergonomic approaches that unravel the responses of the central and autonomic nervous system to sensory conflict and strain, viewed from a unifying, evolutionary active inference perspective, enable us to study and understand underlying neuropsychological mechanisms such as crossmodal integration and attentional dynamics. For instance, sensory conflicts in virtual reality produce a cross-modal “semantic incongruency response” in the EEG signal (Lehser et al., 2024) that can be used to study the degree of fidelity and synchrony that must be achieved to maintain an immersive experience in mixed reality environments, see (Strauss et al. 2024) or general evidence of sensory strain in mobile brain–body imaging studies (Gramann et al., 2011, 2010). In the car-sickness example (Section 3.1), such conflicts may be mitigated through sensory substitution, in which the missing visual information about the vehicle's motion is conveyed via another sensory modality, e.g., see (Schneider et al. 2026) for a virtual-haptics substitution approach. At the same time, the study of sensory conflicts can advance methods to decode human intentions, attentional focus, and affective states, thereby providing a principled foundation for designing human-centered technologies, e.g., in digital operating rooms (Thinnes et al., 2025) or industry 4.0 settings (Trapero et al., 2025). For example, neurotechnology-driven empathetic AI systems (McStay, 2024) are able to “read” human mental and emotional states by integrating signals related to cognition and affect acquired through neurotechnology, see (Alarcao and Fonseca 2017) for EEG, (Naseer and Hong 2015) for fNIRS, and (Flotho and Strauss 2017), (Flotho et al. 2023), and (Bhamborae et al. 2025) for contactless, camera based methods. The availability of this information, and the ability to interpret it correctly, allows these systems to engage with human operators in a context-sensitive and state-adaptive manner, improving human-technology interaction, see also (Gramann et al. 2021) and (Bikson et al. 2024). Understanding the underlying cognitive and affective mechanisms and employing empathetic sensing can support a human-centered neuroergonomic design that might compensate for sensory mismatch and strain, thereby improving human-technology interaction despite increasing demands. For instance, such systems could pre-prioritize information in a context-sensitive manner and select the most appropriate sensory modality for its transfer. In a neuroergonomic digital operating room (see Thinnes et al., 2025), the system might recognize that a surgeon is visually engaged while following a pathway displayed through AR glasses and simultaneously auditorily engaged in conversation with the anesthetist. At the same time, it could detect context-sensitive risks, such as further cutting leading to nerve damage, and therefore choose the somatosensory modality to convey critical information via a haptic vest or smart textiles. Such examples extend to all environments in which digital systems generate large volumes of data, and this information can be harnessed to support humans with their limited attentional resources (see also Trapero et al., 2025). Moreover, Figure 2 (left) and our corresponding arguments also explain the haptic advantage for information transfer in Industry 4.0 and05.0 settings (Trapero et al., 2025), as haptic information transfer occurs within the most sensitive peri-personal space.

An implication of these findings, and the reason we introduce the distinction between core (species typical biases) and mantle (individual human), is that although we already understand certain regularities in human sensory and perceptual processing, current neuroergonomic knowledge is still far from sufficient to determine with confidence which mechanisms are evolutionarily entrenched and which can be reshaped through learning. We use this distinction as a pragmatic conceptual tool to highlight the empirical gaps that must be closed before design choices can be made with confidence. “Core” denotes perceptual and attentional constraints that appear resistant to short term change and that likely reflect genetically anchored operating ranges. “Mantle” denotes experience dependent, context sensitive modulatory processes underpinned by synaptic and neuromodulatory plasticity, attentional gain control, and reweighting of predictive priors; these are the components of perception and attention that can be altered by training, task demands, or changes in ecological statistics. At present the literature does not provide the systematic, causal and longitudinal evidence needed to map specific perceptual or attentional operations onto either category. This is precisely why the distinction is useful: it operationalizes the problem and motivates targeted experiments. A focused research program should therefore identify which features remain stable even after prolonged training, determine which features can be reweighted or habituated through immersive protocols, and map the timescales and transfer conditions for such plasticity. Learning and adaptation span rapid trial by trial tuning of sensorimotor loops, medium term consolidation of attentional routines, and slower representational restructuring, as illustrated in Figure 2 (right). Understanding which phases belong to core or mantle will guide whether systems should route critical information to evolutionarily robust modalities or invest in immersive VR training to teach users to manage sensory streams, habituate to specific conflicts, or reweight modalities for safer and more efficient interaction. And if future research is able to reveal more links between neurodiversity and specific gene variants (the individual genetic differences of the core), predictive power for outcomes of cognitive tasks might be gained. In short, the core–mantle distinction is a forward looking framework that makes visible what we do not yet know and directs research toward actionable knowledge for neuroergonomic design. Within the broader effort of unifying sensory conflict and strain through active inference, this distinction also highlights where future work must clarify how precision allocation, plasticity, and predictive control jointly shape the boundary between species typical constraints and individual level adaptation.

## 6 Conclusions

Future neuroergonomic research should adopt a first-principles, evolution-anchored approach to what environments humans are built for, especially given the growing sensory conflict and sensory strain that characterize the Anthropocene. This requires acknowledging that human perception operates through both core, species-typical constraints and mantle-level, experience-dependent plasticity, which jointly determine how far sensory and attentional mechanisms can be adapted through training or technology in contexts that induce conflict or sustained strain. Design must therefore build on this dual structure, respecting genetically entrenched limits while leveraging modulatory learning processes to scaffold safe adaptation. Within a multimodal active inference framework, we show that sensory conflict and sensory strain can be fruitfully modeled, offering a unified theoretical framework while acknowledging that empirical validation across contexts is still needed. Advances in neurophysiological decoding and attention-aware modality selection may help mitigate these Anthropocene-specific pressures by dynamically prioritizing and routing information. This potentially yields more resilient and effective human–technology interactions that are better aligned with human perceptual and attentional capacities in increasingly complex environments.

## Data Availability

The original contributions presented in the study are included in the article/supplementary material, further inquiries can be directed to the corresponding author.
